# Developing a long‐term follow up service for bariatric surgical patients in the community: Patient and professional perspectives

**DOI:** 10.1002/osp4.658

**Published:** 2023-01-12

**Authors:** Yitka Graham, Ann Fox, Kamal Mahawar, Julie Parrott, Fadi Khalil, Catherine Hayes

**Affiliations:** ^1^ Faculty of Health Sciences and Wellbeing Helen McArdle Nursing and Care Research Institute University of Sunderland Sunderland UK; ^2^ Sunderland Clinical Commissioning Group Sunderland UK; ^3^ Faculty of Psychology University of Anahuac Mexico Mexico City Mexico; ^4^ Bariatric Surgical Unit Sunderland Royal Hospital Sunderland UK; ^5^ Department of Clinical and Preventive Nutrition Sciences Rutgers University New Brunswick New Jersey USA; ^6^ Broadway Medical Practice Springwell Health Centre Sunderland UK

**Keywords:** bariatric surgery, community, General Practice, long‐term follow up, patient perspectives

## Abstract

**Background:**

In the UK, bariatric surgical patients are followed up for 2 years post‐operatively in hospital settings, before being discharged into General Practice for long‐term follow‐up. Presently, there is ambiguous guidance as to what should be included in a community‐based bariatric surgical follow‐up service. The aim of the study was to understand, from both patient and professional perspectives, what is needed to support the long‐term management of bariatric surgical patients in community‐based settings.

**Methods:**

Post‐surgical bariatric patients and General Practice staff were recruited from an area in the UK which has an National Health Service (NHS) hospital providing a high‐volume and established bariatric surgical service. Data was collected through semi‐structured interviews. A thematic analytic framework was used to construct eight themes which illuminated the participants' experiences. The study took place between March and December 2021.

**Findings:**

Thirty participants (14 patients and 16 healthcare professionals) were recruited to the study. The study revealed the lack of a framework for delivery of a long‐term follow up service was frustrating to both patients and professionals. Patient participants reported needing more support, especially dietetic and psychological input, and professionals stated they had little knowledge about bariatric surgical care, and what was needed to provide optimal care, but wanted to provide quality patient care.

**Conclusion:**

Long‐term follow up of bariatric surgical patients is an important issue which needs addressing. This study illuminates both the patient and professional perspectives on developing a pragmatic, community‐based service which meets the needs of patients and considers the need to incorporate such a service into existing infrastructures without adding additional demands on General Practice.

## Graphical abstract

People living with bariatric surgery require long‐term follow up and monitoring to maintain weight‐loss and an improved quality of life. Currently, there is little guidance on service provision in terms of what patients want and need from a service and how this can be integrated into General Practice.

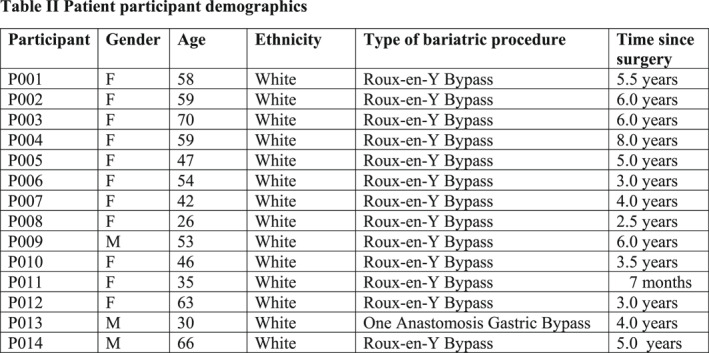

## 1 INTRODUCTION

In the UK, patients who undergo bariatric surgery are followed up for 2 years in Secondary Care before being discharged into General Practice for long‐term follow up. General Practice, sometimes referred as Primary Care or Family Medicine, is defined as a range of non‐acute services provided in community settings such as doctors' offices and community pharmacy offering patient care. Guidance as to what should be provided in a community‐based follow up service gives an overview of the areas to be covered,^1^ guidance on nutritional requirement,^2^, ^3^ and suggestions for managing bariatric patients in the community^4^ have been published, but there is no formal consensus, detailed protocol or which healthcare professionals should be included in this type of follow‐up service and what it should entail.

The knowledge levels of bariatric surgical care amongst many General Practice staff are currently unknown, with research from patients suggesting this may be low.^5^ The bariatric surgical unit at Sunderland Royal Hospital is one of the busiest units in the UK, and it follows that there are a correspondingly high number of bariatric surgical patients living within Sunderland who require long—term support and monitoring in community settings.

With current high demands on General Practice in the UK,^6^ the logistics of developing a pragmatic, viable framework to embed and sustain a long term follow up service for the monitoring and support of people living with bariatric surgery need to be scoped and established. Studies have shown that Community Pharmacists, who have diverse roles in General Practice, possess specialist expertise and knowledge, for example, pharmacokinetics, which are important to bariatric surgical care, as the effects of many medications and supplements are altered after bariatric surgery, given the malabsorptive and restrictive elements of procedures.^7^, ^8^, ^9^ Nearly 90% of the UK population has a community pharmacy accessible in a 20 min walk,^10^ community pharmacies have longer opening hours than other healthcare providers, do not require an appointment, and 90% have private consulting rooms.^11^ Pharmacists are also working more closely with General Practice as part of the UK National Health Service's (NHS) Long Term Plan^12^ to develop and deliver local services based on patient needs.^13^ The role of Community Pharmacy may have potential to contribute to supporting follow‐up care for people who have undergone bariatric surgery, but like other General Practice professionals, the roles are currently not articulated. Any community‐based bariatric follow‐up service needs to be developed with the voices of both services users as well as healthcare professionals, ensuring that the service would be pragmatic to deliver and meet the needs of patients. Studies carried out from the patient perspective in bariatric surgical care^5^, ^7^ have been helpful in providing insight into care and influencing healthcare decision‐making.

The aim of the study was to understand, from both patient and professional perspectives, what is needed to support the long‐term management of bariatric surgical patients in General Practice settings.

## 2 MATERIALS AND METHODS

A qualitative methodological framework underpinned the study. Qualitative methods are useful when trying to understand peoples' thoughts, experiences and feelings toward the subject under investigation.^14^ Data from participants is often collected in their “natural environment” for example, a surgery for a general practitioner (GP), and a domestic or other setting for a patient, in order that the participant feels comfortable taking part in the study.^15^

Two cohorts of participants were recruited to the study using clear inclusion and exclusion criteria (see Table 1). Firstly, patients who had undergone bariatric surgery at Sunderland Royal Hospital were identified from clinic lists and approached in writing with study information including a letter of invitation, a participant information sheet, a consent form, and a reply‐paid envelope. All participants were informed they could contact Yitka Graham if they had any queries before making their minds up about whether to take part or not. Owing to the geographical specificity of the bariatric surgical service, and local General Practice settings who serve most patients who underwent bariatric surgery there, the inclusion criteria was limited to meet the aims of the study.

**TABLE 1:** Inclusion/exclusion criteria

**Inclusion criteria**	**Exclusion criteria**
**1. Healthcare professionals**	
Staff working in General Practice within the city of Sunderland (full and part‐time)	Staff working in General Practice outside the city of Sunderland
Ability to provide consent	Staff who were retired, off sick, or unable to provide consent
**2. Patients**	
People who have undergone bariatric surgery procedures (Roux‐n‐Y gastric bypass, one anastomosis gastric bypass, sleeve gastrectomy)	People who are on waiting lists or have not undergone bariatric procedures
Primary or revisional bariatric procedure	People living outside Sunderland
People living in the city of Sunderland	Inability to provide informed consent
Ability to provide informed consent	

Healthcare professionals working in General Practice across Sunderland were identified by a register of GP practices and approached in writing. Potential participants who wanted to discuss the study further, along with those who wished to take part contacted Yitka Graham, who answered any questions and for those who wished to take part, took informed consent prior to data being collected.

**FIGURE 1:**
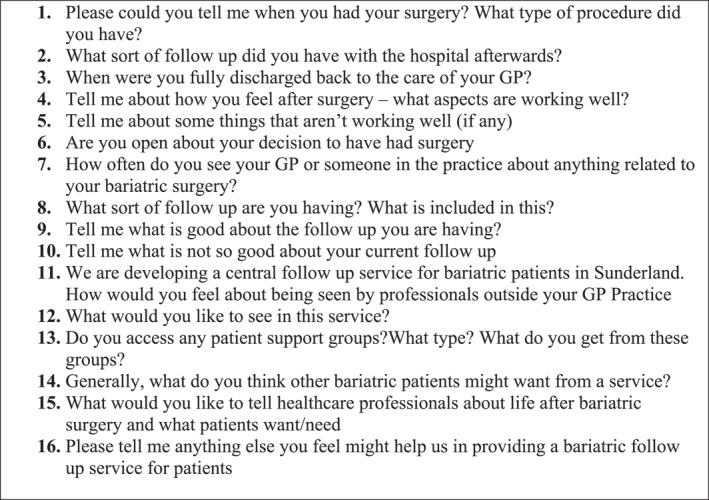
Topic guide for patient participants.

**FIGURE 2:**
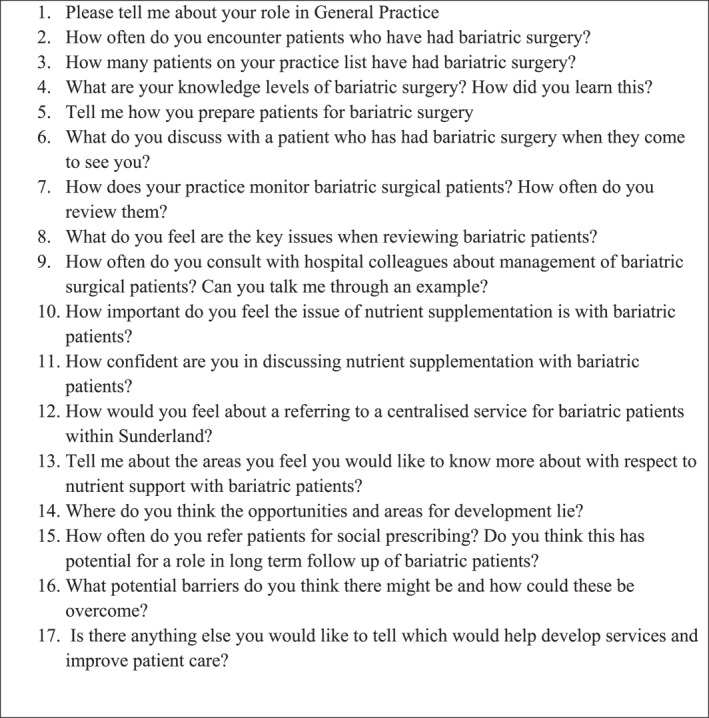
Topic guide for healthcare professional participants.

Data was collected through individual, interviews with participants (using video‐conferencing to adhere to social distancing guidelines), using a topic guide (see Figures 1 and 2) to facilitate discussion.^14^ Interviews were audio recorded, anonymized and transcribed verbatim by an approved transcription company. A £10 Amazon® voucher was offered to all participants.

Data were analyzed through a data thematic analytic framework, informed by the methods of Braun and Clarke^16^ and Robson.^17^ Thematic analysis is a qualitative methodology which allows patterns or themes to be constructed from collected data, with themes allowing exploration of the parts of the situation, allowing a greater understanding of the whole.^18^ The patterns, or themes constructed from the data present an interpretation of the situation under investigation, with the thematic analysis informing recommendations and scope for further research. Transcripts were read by the research team to familiarize themselves with the data, followed by each person identifying codes, or areas that were felt to be relevant to answering the research question. These were then discussed amongst the team, with an initial set of themes produced. These themes were discussed and further refined, with a consensus reached on the final eight themes. The data analysis was enhanced by a social constructivist perspective, which asserts that reality is shaped through interactions with others and influenced by life experiences, social norms and values.^19^ Using this perspective helped to understand the perceptions of the social constructs of roles of patient and professionals which would assist to further illuminate findings.

This study received favorable ethical approval from the UK Health Research Authority and the University of Sunderland Research Ethics Group.

## 3 RESULTS

There were 30 participants recruited to the study, comprising patients (*n* = 14) (see Table 2) and healthcare professionals (*n* = 16) (see Table 3). Recruitment took place from July to October 2021. There were eight themes constructed from the data, four with patient participants and four with healthcare professionals. Of the 14 patient participants, 11 were female (78%) and 3 were male (22%). The mean age was 50, ranging from 26 to 70 years of age. Time from surgery ranged from 7 months to 8 years (average time 4.4 years).

**TABLE 2:** Patient participant demographics

Participant	Gender	Age	Ethnicity	Type of bariatric procedure	Time since surgery
P001	F	58	White	Roux‐en‐Y bypass	5.5 years
P002	F	59	White	Roux‐en‐Y bypass	6.0 years
P003	F	70	White	Roux‐en‐Y bypass	6.0 years
P004	F	59	White	Roux‐en‐Y bypass	8.0 years
P005	F	47	White	Roux‐en‐Y bypass	5.0 years
P006	F	54	White	Roux‐en‐Y bypass	3.0 years
P007	F	42	White	Roux‐en‐Y bypass	4.0 years
P008	F	26	White	Roux‐en‐Y bypass	2.5 years
P009	M	53	White	Roux‐en‐Y bypass	6.0 years
P010	F	46	White	Roux‐en‐Y bypass	3.5 years
P011	F	35	White	Roux‐en‐Y bypass	7 months
P012	F	63	White	Roux‐en‐Y bypass	3.0 years
P013	M	30	White	One anastomosis gastric bypass	4.0 years
P014	M	66	White	Roux‐en‐Y bypass	5.0 years

**TABLE 3:** Healthcare professional demographics

Participant	Gender	Age	Ethnicity	Profession
H001	F	31	White	Community pharmacist based in one General Practice
H002	F	56	White	Pharmacy services commissioner
H003	F	23	White	Community pharmacist based across three General Practices
H004	M	25	White	Community pharmacist based across three General Practices
H005	F	52	White	Practice nurse
H006	F	47	White	Practice nurse
H007	F	46	White	Practice nurse
H008	M	56	White	General practitioner
H009	M	37	White	General practitioner
H010	F	31	White	Community pharmacist based in General Practice
H011	F	42	British African	General practitioner
H012	F	39	British Asian	Community pharmacist based in General Practice
H013	F	26	White	Practice nurse
H014	F	37	British African	Community pharmacist based in General Practice
H015	M	56	White	General practitioner
H016	M	47	British Asian	General practitioner

Analysis of the data revealed four themes which are discussed in detail and supported by in vivo quotes to illuminate the participants' experiences.

### 3.1 Theme 1 (patients) feeling that community healthcare professionals do not understand what patients go through after surgery

Many patients reported feeling that healthcare professionals in the community did not seem have an understanding of what people go through when they have surgery. This appeared to come out in clinical encounters:

I don't think they [GPs] really understand what people go through when they have bariatric surgery, they don't seem to know enough.

(Participant 8)

Many reported that initiation of follow‐up care is through the patient and not the practice:

I'm the one who needs to instigate my bloods to be done once a year. Even though the hospital sends a letter out saying they are due, I never get a phone call to say they need doing….it is never, ever, mentioned. I also haven't been weighed for years.

(Patient Participant 7)

### 3.2 Theme 2 (patients) needing longer term support to ensure optimal wellbeing

The majority of the patient participants acknowledged the need for longer term support, for weight‐loss and wider health and wellbeing, with mental wellbeing stated as an area where support was not clear nor apparent:

The main thing is just to be supportive. I know time is an issue but don't try and rush through. Let us talk, let us tell them [healthcare professionals] how it's going. It's not always about the numbers. Yes, we are excited that we are losing weight, but it's not the number that are changing, it's our whole mentality, our whole person.

(Patient Participant 16)

### 3.3 Theme 3 (patients) needing a multidisciplinary approach to support and care

Patient participants reported needing a biopsychosocial approach to long‐term follow‐up, being able to draw upon the support of a range of expertise from clinicians:

*Psychology and mental health*:

I think follow‐up needs to focus on the mental health side of things. To get a gastric bypass you need to go through a psychologist, and you have to deal with things. After the operation, that completely stops, and although you might be fit and ready, the ‘how am I going to do this’ feeling happens. I know so many people who have had it done, and they have said they are suffering mentally because they can't cope with the changes, getting divorced, hating the way their body looks…. I think follow‐up needs to be based on, or fully focused on people's mental health afterwards.

(Patient Participant 12)

*Dietitians*:

I think there should be more help for the diet type of thing because you're sort of just chucked back in [after surgery] and that's it. There should be more help in how to maintain diet. I've got books and everything, but I don't think there's much follow up on how to maintain your weight, not much dietitian support available. I mean, you jump through all the hoops to get to surgery, then afterwards, there's not much.

(Patient Participant 10)

### 3.4 Theme 4 (patients) coping with the pandemic in the context of living with a bariatric‐surgically altered body

Analysis found that the pandemic had an acknowledged impact on people living with a bariatric‐surgically altered body, which needs to be taken into consideration as the temporality of the pandemic is as yet unknown and is set to continue for the foreseeable future. Many patient participants discussed the impact of the pandemic on their post‐surgical journeys.

Patient participants felt that the social distancing measures and national lockdown because of the pandemic had impacted on their post‐surgical experiences, which they found difficult to deal with:

I think half my problem is habit. Because of COVID‐19 I have to work from home now, literally steps away from the fridge and my head is constantly in the fridge. And drinking…. drinking alcohol over the pandemic, over lockdowns and things have massively increased for me. To the point that I've not had to reel in and say ‘no, we're not drinking during the week, even though I don't have to get in the car and drive to work’. I was a picker before the surgery and now getting back into that habit has not been good’.

(Patient Participant 2)

Of the 16 healthcare professionals recruited to the study, 11 were female (69%) and 5 were male (31%). The healthcare professions represented in this study were community pharmacists (working in a variety of community‐based roles) (44%, *n* = 7), Practice Nurses (25% *n* = 4), and GPs (31%, *n* = 5). The mean age of the healthcare professional participants was 40 years, ranging from 27 to 56 years of age. Analysis of the health care professional data revealed four further themes.

### 3.5 Theme 5 (professionals) reporting a lack of knowledge about bariatric surgery and post‐surgical needs of patients

Many healthcare professionals stated they had not received any formal training or education on bariatric surgical care for patients:

I know they do gastric sleeves and things, but I don't know the procedures well enough to be able to sit down and discuss this with patients. I learned by reading and when I see patient who have had surgery, I ask them and get them to explain this to me. So, I only know a bit from the patients I've seen and what I've read.

(Healthcare Professional 14)

### 3.6 Theme 6 (professionals) acknowledging the lack of a formal process for managing post‐bariatric surgical patients in General Practice and community healthcare settings at present

There is no protocol as such. We get a letter from the hospital highlighting what they are expecting the practice to follow up on and to do. Starting supplements, things like that, but no protocols as such. If a patient contacts us and says ‘I have had surgery on such and such a date and I was told to contact, you with regards to this’. We would bring them in every twelve weeks for B12 injections and things like that, and also to monitor other ongoing health issues such as hypertension, we'd be reviewing them for that anyway, but no specific bariatric protocol’.

(Healthcare Professional 8)

### 3.7 Theme 7 (professionals) identifying patient‐reported issues and managing expectations

Healthcare professionals felt that many patients needed to understand that there was more to bariatric surgery than just weight loss, and expectations following surgery needed to be managed:

They [patients] think their life is going to be complete when they lose weight, but actually, that's just one part of it…that you've still got to make an effort to be fit. Losing weight doesn't mean you're fit and it doesn't mean you're happy. And I think there is that expectation that once they lost the weight, their life will be complete and everything will be fine….it takes a couple of years down the line for them to realise that is not necessarily the case.

(Healthcare Professional 14)

### 3.8 Theme 8 (professionals) considering how patients could be supported in the community

The expertise of other healthcare professionals could be utilized to support GPs:

Pharmacists are in a good position to support. When we do the structured medication review we take a more holistic approach, we explore their lifestyles, BMI etc…we explore mood, sleep, bowels and what they've been swallowing…I have referred a few bariatric patients for social prescribing.

(Healthcare Professional 11)

To understand the current situation further, and to illuminate areas of best practice, two examples of where patient participants discussed exemplars of where follow‐up encounters had been very positive:

I have a very chatty nurse that does my B12 injections because she's always talking about how things are going. I said, ‘I'm doing more walking now’ and she will remember and ask, ‘have you been walking anywhere?’ and I'm like how can she remember all of this?

(Patient Participant 16)

Two examples of areas where current provision could be improved were reported by patient participants. The first highlights encounter reports weight bias by the healthcare professional which made the patient feel upset:

I was poorly and went to the chemist and she did my blood pressure, and it was low. The pharmacist said, ‘it's really because of your dramatic weight loss, you need to come off some of your blood pressure tablets’ and then said, ‘oh well, at the end of the day you went on the blood pressure tablets because of your weight in the first place, which made me feel like crap’.

(Patient Participant 8)

The second example highlights the patient‐reported need for improvement in current follow‐up provision in the community:

The aftercare needs to be improved a lot more than what it is. They [healthcare professionals] need to be aware that it is not an easy thing for people, and they can't just be left to it straight after. I think there needs to be a lot more aftercare, checking not just on weight but to make sure are eating right and how they feel about it and if they are struggling. There's not much aftercare in my opinion, but other people have said theirs was brilliant.

(Patient Participant 9)

## 4 DISCUSSION

This was the first study to explore the patient and healthcare professionals' perceptions of long‐term follow up of bariatric surgical patients in the community in Sunderland. Sunderland has a higher than average rate of adult obesity in England.^20^ The bariatric surgical service at South Tyneside and Sunderland NHS Foundation Trust is one of the highest volume centers in England, with the majority of patients undergoing bariatric surgery living in the local area, accessing local General Practices for follow‐up. In line with current guidance, patients who have undergone surgery are generally followed up by Secondary Care for 2 years before being discharged into General Practice for long‐term follow up and monitoring.^1^ As stated earlier, several articles offering advice and guidance for community follow‐up have been published, but consensus on a formal criteria and process is lacking.

Both patient and healthcare professionals reported that the lack of a formal long‐term follow up service for bariatric patients was frustrating and difficult in terms of optimal support and care. Common to both cohorts of participants was the identified need for a multidisciplinary approach to long‐term follow up, with a particular emphasis on identifying and supporting mental health needs. Of particular interest was that many of the healthcare professionals were unaware of how many bariatric patients they had in their practice and how often they presented. The variance in response suggests this information is not readily available, which we argue is needed to understand not only the number of patients, but why they are presenting and what their needs are.

The healthcare professionals who took part in this study reported low levels of knowledge about bariatric surgical procedures and optimal care of post‐bariatric patients. Literature shows this is a common phenomenon in General Practice.^21^, ^22^ The patient participants in this study felt that healthcare professionals did not understand what patients wanted and needed after surgery, this has also been identified in studies.^23^ Patient participants did not blame individual healthcare professionals for this, our study showed that this was interpreted as a wider, systems issue.

There was a paucity of published studies examining patient and healthcare professionals' experiences of utilizing and providing bariatric patient support in General Practice settings. Literature has shown that the lack of formal guidance for long‐term follow up of bariatric patients is resulting in patients not being able to either access or receive optimal care. As study of over 3000 patients in the UK found that patients were not receiving the recommended nutritional monitoring in the community.^24^ A retrospective analysis of 385 patients found that patients who attended follow‐up clinics were more likely to have better excess weight loss and total weight loss than those who did not access follow up services.^25^

A study on post‐surgical outcomes with 169 sleeve gastrectomy (second most commonly performed procedure in the UK) found that after 5 years, only half the patients had achieved more than 50% of their excess weight loss, with physical activity, eating patterns, and eating pathologies influencing weight outcomes,^26^ clearly supporting the need for dietitian support as found in this study. The need for psychological support as part of long‐term follow‐up was identified by both patients and healthcare professionals.

The patient transition from Secondary Care to General Practice and community care after 2 years may be better supported with a coordinated approach, where General Practice is involved during the handover, so follow‐up is coordinated in collaboration with the bariatric multidisciplinary team, for example, surgeons, the dietitian and mental health professionals.^27^ It is noted that the multidisciplinary team approach to bariatric care is an established concept in Secondary Care,^1^, ^28^ but there is no parallel or remotely similar model in General Practice. A qualitative study of GPs in Australia found that greater clarity of their role in bariatric patient follow up and the need to work with Secondary Care to optimize patient care^29^ supports the findings of our study, and may suggest that this phenomenon is happening elsewhere.

The need for greater understanding of the experiences of people living with a bariatric surgically altered body and for this to be considered and incorporated into follow up patient care has been found in other qualitative studies,^5^, ^30^, ^31^ highlighting the need for a multidisciplinary management of patients and greater mental health provision,^32^ reflects the findings of the patient cohort of our study.

Studies have found that post‐operative bariatric care and patient compliance has been adversely affected by the pandemic.^33^, ^34^ The data for this study was collected in 2021, a year into the pandemic, which means that any potential impact of COVID‐19 for example, social distancing measures and other impact on post‐surgical bariatric patient care was captured, and incorporated into recommendations for future planning of service provision. Many healthcare professionals reported seeing fewer bariatric surgical patients since the start of the pandemic.

The limitations of this work are that it was undertaken in a specific area of the UK, and the population demographics and community healthcare provision may be different in other areas of the UK and in other countries. There were also a high number of White participants in our study, but this is reflective of the local population demographics. We had more female patient participants than male in the study, but it is evidenced that more females than males undergo bariatric procedures^35^ The strengths of this study were that both patients and healthcare professionals were represented, so comparisons could be made to offer a balanced perspective to inform recommendations and future service development of community‐based follow‐up services for bariatric patients.

There are several recommendations to consider when developing this service in the future. The self‐reported healthcare professionals' lack of knowledge of bariatric surgical procedures and of the post‐surgical psychosocial impact needs to be addressed through formal education and training, approaching this from a biopsychosocial perspective to capture a diverse range of factors influencing health and wellbeing. This may be incorporated into routine continuous professional development. A greater emphasis on the mental health and wellbeing of post‐bariatric patients needs to be formalized as part of routine bariatric care. Healthcare professionals such as psychologists working in community settings may be best placed to address this, but it is acknowledged there is a current workforce shortage with psychologists.^36^ A multidisciplinary model of care in the General Practice needs to be established going forward, following a model like that in bariatric surgical clinics. Core members of General Practice multidisciplinary teams should include dietitians, psychologists, pharmacists, and a GP or Practice Nurse or Nurse Consultant with a designated specialist interest in bariatrics. This model should be piloted and evaluated, with a view to using this as a framework to develop a model of care for long‐term follow‐up and support of bariatric patients. The role of the community pharmacist should be further explored to ascertain if bariatric surgical care could be built into routine structured medication reviews which are carried out annually in the UK.

Bariatric surgery a life‐changing and often life‐saving intervention for weight‐loss and comorbidity improvement. Prioritizing community‐based support and follow‐up, which is developed in a manner which improves quality of life for patients and streamlines care provision in General Practice is crucial.

## CONFLICT OF INTEREST

No authors have any conflict of interest to disclose.
